# Daptomycin-Associated Diarrhea: A Case Report and Review of the Literature

**DOI:** 10.7759/cureus.26135

**Published:** 2022-06-20

**Authors:** Abdalaziz M Awadelkarim, Isra Idris, Muhammad Abdelhai, Ahmed Yeddi, Eltaib Saad, Rashid Alhusain, John Dayco, Mohammed Ali, Lubna Salih

**Affiliations:** 1 Internal Medicine, Wayne State University Detroit Medical Center, Detroit, USA; 2 Pediatrics, Woodhull Medical Center, New York, USA; 3 Internal Medicine, Saint Francis Hospital, Evanston, USA

**Keywords:** clostridioides difficile -associated diarrhea, adverse drug events, antibiotics-associated diarrhea, daptomycin-associated diarrhea, daptomycin

## Abstract

Antibiotic-associated diarrhea (AAD) describes any unexplained diarrhea associated with the use of antibiotics. AAD develops through diverse mechanisms, ranging from pharmacologic effects on gut motility to disturbance of the function and carbohydrate metabolism of the indigenous intestinal flora and overgrowth by pathogenic micro-organisms. Clostridioides difficile-associated diarrhea (CDAD) is a subset of AAD; however, it accounts only for a small percentage of diarrhea caused by antibiotics. Diarrhea has been reported as a side effect of daptomycin use, nevertheless, it's thought to be mild and carries significantly less risk of diarrhea than other alternative treatments of S. aureus bacteremia, i.e., vancomycin or cefazolin. The authors present an interesting case of daptomycin-associated diarrhea presenting with a protracted and severe course. Patient symptoms didn’t improve with empiric Clostridioides difficile therapy and CDAD testing was negative. Diarrhea promptly resolved after discontinuation of daptomycin. Furthermore, a thorough literature review was conducted and discussed in this article to raise awareness of this under-recognized complication. Clinicians should be mindful of daptomycin-associated diarrhea along with its presentation and treatment. Further studies are needed to identify the pathophysiology of daptomycin-associated diarrhea and other forms of AAD. Understanding their mechanism could help prevent, treat, and reduce the significant medical costs associated with antibiotic adverse events.

## Introduction

Diarrhea is a common adverse event, accounting for about 7% of all drugs' adverse effects. More than 700 drugs have been implicated in causing diarrhea; commonly involved classes are antimicrobials, prostaglandins, colchicine, nonsteroidal anti-inflammatory drugs, laxatives, magnesium-, lactose- or sorbitol-containing products, antineoplastics, antiarrhythmic medications, and cholinergic agents [1]. Several pathophysiological mechanisms are involved in drug-induced diarrhea, including the alternation of gut bacterial flora, osmotic diarrhea, secretory diarrhea, exudative diarrhea, protein-losing enteropathy, shortened transit time, malabsorption, or maldigestion of fat and carbohydrates [1].

Antibiotic-associated diarrhea (AAD) describes any unexplained diarrhea associated with the use of an antibiotic. AAD develops through diverse mechanisms, ranging from pharmacologic effects on gut motility to disturbance of the function and carbohydrate metabolism of the indigenous intestinal flora and overgrowth by pathogenic micro-organisms [2]. Rates of AAD vary from 5 to 39% depending upon the specific type of antibiotic [3]. Clostridioides difficile-associated diarrhea (CDAD) is a subset of ADD; however, it accounts only for a small percentage of diarrhea caused by antibiotics (10-20%). AAD can be caused by multiple other organisms, including Clostridium perfringens, Staphylococcus aureus, Salmonella spp., and Candida. Some antibiotics are more likely to cause non-CDAD, such as erythromycin and the penicillin class [2,4]. Klebsiella oxytoca was identified as the causative agent of antibiotic-associated hemorrhagic colitis (AAHC), a severe subset of AAD [5]. Understanding the different mechanisms that cause AAD and prevent it could potentially improve medical care and significantly reduce medical cost.

Clinically, two patterns of ADD are typically seen: acute diarrhea, appearing within the first few days of initiating an antibiotic regimen, and chronic diarrhea, lasting more than three to four weeks and can appear multiple weeks after the start of drug therapy. Both can be severe and poorly tolerated. The disease spectrum ranges from mild diarrhea to pseudomembranous colitis and AAHC [1,2]. Diarrhea has been reported as a side effect of daptomycin use in 4-7% of cases. It's thought to be mild and carries significantly less risk of diarrhea than other alternative regimens for the treatment of S. aureus bacteremia, i.e., vancomycin or cefazolin [6]. Severe and protracted diarrhea associated with daptomycin use has never been reported in the literature. We present a novel case of daptomycin-associated diarrhea presenting with protracted and severe non-bloody diarrhea, which improved within 48 hours of stopping the medication. Furthermore, a thorough literature review was conducted and presented in this case report to raise awareness of this under-recognized complication.

## Case presentation

A 53-year-old female with past medical history of oropharyngeal mucocutaneous T-cell lymphoma in remission presented to the hospital with severe watery diarrhea (nine to 12 motions/day) for five days. The patient was discharged one week earlier for admission with sepsis secondary to mediport-associated methicillin-resistant Staphylococcus aureus (MRSA) bacteremia, complicated by tricuspid valve infective endocarditis. She was initially treated with intravenous vancomycin. The isolated strain showed minimal inhibitory concentration (MIC) of 2 mcg/ml to vancomycin. Phoenix system was utilized for assessment of MIC and confirmed with Etest. Two days before discharge, she was switched to daptomycin. After clearance of bacteremia, a peripherally inserted central line (PICC) was placed. The patient was discharged home on daptomycin 6 mg/kg per day with a plan to complete a six-week course of antibiotics.

**Figure 1:**
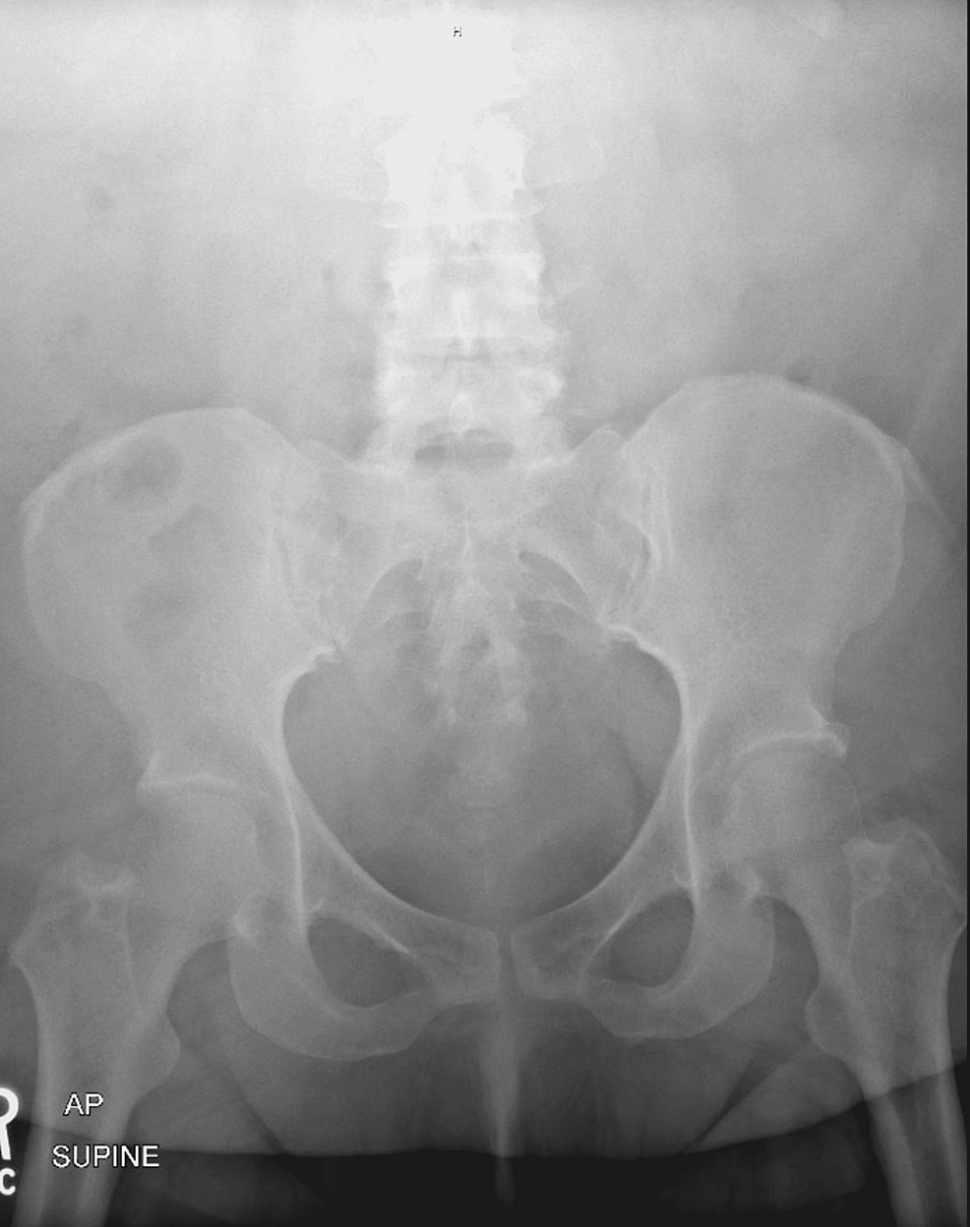
Abdominal X-Ray showed non specific gas pattern

Shortly after going home, the patient started to experience profuse watery diarrhea, associated with tenesmus and urgency. It progressively increased in frequency to nine to 12 motions/day. She endorsed nausea but denied vomiting, melena, hematochezia, abdominal pain, or fever. The patient denied any recent travel, sick contact, NSAID intake, or similar history in the past. On presentation, she was afebrile and normotensive-tachycardic with heart rate of 104 beats per minute. The abdomen was soft, non-distended, and non-tender on exam. The guaiac exam was negative. Laboratory findings showed white blood count 9.7 k/𝜇L (normal range 3.3-10.7 k/𝜇L), absolute neutrophil count 7.8 k/𝜇L (normal range 1.6-7.2 k/𝜇L), Hgb 13.8 g/dL (normal range 12.1-15.0 g/dL), mean corpuscular volume (MCV) 88 fL (normal range 80-100 fL), platelets 312 k/𝜇L (normal range 150-400 k/𝜇L), blood urea nitrogen 32 mg/dL (normal range 9-25 mg/dL), creatinine 1.2 mmol/L (normal range 0.5-1.2 mmol/L), lactic acid 2.4 mmol/L (normal range 0.5-2.2 mmol/L) and C-reactive protein (CRP) 34 mg/dL (normal range 0-7 mg/dL). Liver function tests and creatine phosphokinase were within normal limits. The abdominal X-ray was grossly normal and showed a nonspecific gas pattern (Figure 1). Laboratory results on presentation are shown in Table 1. 

**Table 1:** Summary of initial laboratory results

Laboratory test	Patient's result	Reference range
White Cells Count (WCC)	9.7 k/ 𝜇L	3.3-10.7 k/ 𝜇L
Absolute Neutrophil Count	7.8 k/𝜇L	1.6-7.2 k/ 𝜇L
Neutrophils Percentage	80%	40-65%
Hemoglobin	13.8 g/dl	12.1-15.0 g/dL
Mean corpuscular volume (MCV)	88 fL	80-100 fL
Platelets	312 k/ 𝜇L	150-400 k/ 𝜇L
Serum Sodium	138 mmol/L	133-144 mmol/L
Serum Chloride	99 mmol/L	98-107 mmol/L
Serum Potassium	3.4 mmol/L	3.5-5.2 mmol/L
Serum Bicarbonate	21 mmol/L	21.0-28.0 mmol/L
Blood Urea Nitrogen (BUN)	32 mg/dL	9.0-25.0 mg/dL
Serum Creatinine	1.2 mmol/L	0.5-1.2 mmol/L
Lactic Acid	2.4 mmol/L	0.5-2.2 mmol/L
C-reactive protein (CRP)	34 mg/dL	0-7 mg/dL

Initially, the diarrhea was thought to be secondary to CDAC, and due to her significant symptoms, she was started empirically on oral vancomycin, 150 mg, orally, four times daily (QID). During this time, daptomycin for MRSA bacteremia and infective endocarditis was continued. Nevertheless, initial stool studies, including stool culture, ova and parasite, and Clostridioides difficile toxin PCR were negative. Blood testing for antibodies for immunoglobulin A (IgA) tissue transglutaminase was undetectable. At that time, the team thought that Clostridioides difficile PCR (sensitivity, 86%; specificity, 97%) result could represent a false negative [7]. Oral vancomycin dose was increased to 500 mg QID, and metronidazole was added without any improvement in her symptoms and diarrhea frequency. Eventually, a repeat Clostridioides difficile test was negative. Blood testing for antibodies for immunoglobulin A (IgA) tissue transglutaminase was undetectable. Antimotility medications (loperamide) were tried with no improvement in patient symptoms.

Given the lack of response, medication-associated diarrhea was suspected as the patient started developing diarrhea two to three days after she was started on IV daptomycin. After discussion with infectious disease services and the fact that the diarrhea is becoming more debilitating, a trial of stopping daptomycin and switching to linezolid was attempted. Within 48 hours of stopping daptomycin, the patient's diarrhea improved significantly, and she finished her remaining course of antimicrobials for infective endocarditis using linezolid. At that time, the diagnosis of daptomycin-associated diarrhea was considered the likely cause of her case, given the solid temporal relation of IV daptomycin use and the patient's diarrhea. She was discharged home in stable condition.

## Discussion

Daptomycin is an acidic cyclic lipopeptide antibiotic that was the first of its class to treat gram-positive resistant pathogens [8]. It is a naturally occurring compound found in the soil saprotroph Streptomyces roseosporus. Daptomycin exerts its bactericidal effect by disrupting multiple aspects of bacterial cell membrane function, causing rapid depolarization of membrane potential, that leads to the intracellular inhibition of DNA, RNA, and protein synthesis [9,10].

Daptomycin is only active against gram-positive bacteria. It has broad-spectrum coverage against resistant organisms, including vancomycin-resistant enterococci (VRE) and methicillin-resistant Staphylococcus aureus (MRSA) [11]. Daptomycin is FDA approved for use in adults in the United States for S. aureus bacteremia and right-sided S. aureus endocarditis caused by methicillin-susceptible and methicillin-resistant isolates. Additionally, it is indicated for use in complicated skin and skin structure infections (cSSSI) caused by susceptible isolates, including MRSA, VRE, Streptococcus pyogenes, Streptococcus agalactiae, and Streptococcus dysgalactiae subsp. equisimilis (SDSE). Half-life is eight to nine hours in healthy adults with normal renal function. Duration of therapy is seven to 14 days of IV daptomycin 4 mg/kg once daily in the setting of cSSSI. Whereas, in the setting of S. aureus bacteremia and right-sided S. aureus endocarditis, a four- to six-week course of IV daptomycin 6 mg/kg once daily is recommended [6,12].

Daptomycin hasn't been proven to be superior to vancomycin in treating MRSA endocarditis. However, it is considered the drug of choice for treating MRSA endocarditis in the settings of strains with a vancomycin MIC of 2μg/mL, similar to our case. Additionally, it's the drug of choice in the setting of heterogeneous vancomycin-intermediate S. aureus (hVISA) phenotypes and for glycopeptide-intermediate S. aureus (GISA) strains [13]. Daptomycin is inactivated by avidly binding to pulmonary surfactants. Therefore it should not be used for the treatment of pneumonia [14]. Other limitations include left-sided infective endocarditis due to S aureus; the clinical trial in adults with S. aureus bacteremia included limited data from patients with left-sided infective endocarditis; outcomes in these patients were poor [13,15]. Additionally, there have been no studies in patients with prosthetic valve endocarditis. Daptomycin is not recommended in pediatric patients less than one year old due to the risk of potential effects on muscular, neuromuscular, and/or nervous systems (either peripheral and/or central) observed in neonatal dogs [12].

The most widely reported adverse reactions include injection site reactions, headaches, and mild gastrointestinal disturbances (nausea, vomiting and diarrhea) [6,16]. Patients are monitored for rhabdomyolysis and muscle toxicity (myopathy). More frequent monitoring is recommended in patients with renal impairment and/or those receiving concomitant HMG CoA (3-hydroxy-3-methyl-glutaryl-coenzyme A) reductase inhibitors (statins) [17]. Eosinophilic pneumoniae and peripheral neuropathy have been described in the association of daptomycin therapy. Eosinophilic pneumonia generally occurs two to four weeks after initiating therapy and may reoccur with re-exposure to daptomycin [18].

Diarrhea has been reported as a side effect of daptomycin use in 4-7% of cases. CDAD has been described in association with daptomycin use. The incidence of CDAD has been observed greater than two months after treatment with daptomycin. Daptomycin-associated diarrhea is thought to be mild and carries significantly less risk of AAD than the standard of care (SOC) for S. aureus bacteremia [6,12,18]. Arrieta et al. randomized 55 children to daptomycin and 27 to SOC (primarily vancomycin or cefazolin); 90% had S. aureus. In both groups, 15% of patients had drug-related adverse events, mainly diarrhea (4% daptomycin, 8% SOC) and increased creatine phosphokinase (4% daptomycin, 0% SOC) [6]. Severe and protracted diarrhea associated with daptomycin use without CDAD has rarely been described before in the literature.

In our case, the patient developed frequent large, non-bloody, loose bowel movements per day. The review of systems was negative for fever, melena, hematochezia, and abdominal pain. Diagnostic laboratory workup was nonrevealing. Tests sent included a comprehensive metabolic and complete blood count with differential. Further workup came back negative for Clostridioides difficile infection. Stool testing for Giardia, ova, parasites, and stool culture was negative for any culprit. Blood testing for antibodies for IgA tissue transglutaminase was undetectable. The patient continued to have severe diarrhea despite multiple various interventions. Moreover, a trial of antimotility medications (loperamide) was tried but to no avail. After discussion with the patient, a decision was made to switch to daptomycin, and diarrhea improved within 48-72 hours. 

Chronologically there was a strong temporal relationship between the initiation of daptomycin and the development of persistent severe diarrhea. The prompt cessation of symptoms after stopping the medication supports daptomycin as the cause of diarrhea in our case. Moreover, our patient's score falls into the "probable" classification according to the Naranjo probability score (Table 2) [19]. This indicates a probable relationship between diarrhea and daptomycin therapy, given the temporal association without any alternative justification. As with any other form of AAD, it could be explained by diverse mechanisms, including direct pharmacologic effects on gut motility, disturbance of the function and carbohydrate metabolism of intestinal microbiota, and overgrowth of pathogenic micro-organism other than Clostridioides difficile [2]. Nevertheless, given the improvement of diarrhea in 48-72 hours of stopping the antibiotic, the low yield of stool culture (2.4% to 32%) [20], and the fact that associated neutrophilic leukocytosis and elevated CRP were present, we do believe that the diarrhea was likely related to a non-Clostridioides difficile AAD. Another alternative explanation would be a direct pharmacological effect of the medication on gut function. 

**Table 2:** Adverse Drug Reaction Probability Scale (Naranjo algorithm).

Question	Yes	No	Score	Patient Score
1. Are there previous conclusive reports on this reaction?	+1	0	0	+1
2. Did the adverse event appear after the suspected drug was administered?	+2	-1	0	+2
3. Did the adverse event improve when the drug was discontinued, or a specific antagonist was administered?	+1	0	0	+1
4. Did the adverse event reappear when the drug was readministered?	+2	-1	0	0
5. Are there alternative causes that could on their own have caused the reaction?	-1	+2	0	+2
6. Did the reaction reappear when a placebo was given?	-1	+1	0	0
7. Was the drug detected in blood or other fluids in concentrations known to be toxic?	+1	0	0	0
8. Was the reaction more severe when the dose was increased or less severe when the dose was decreased?	+1	0	0	0
9. Did the patient have a similar reaction to the same or similar drugs in any previous exposure?	+1	0	0	0
10. Was the adverse event confirmed by any objective evidence?	+1	0	0	+1
Total Score ≥9: Definite. 5-8: Probable. 1-4: Possible. ≤0: Doubtful.				Total Score: 8

In summary, this report highlights a probable causation between daptomycin and non-Clostridioides difficile-related severe diarrhea, based on exclusion of other etiologies and resolution of diarrhea shortly after discontinuation of daptomycin. However, pathophysiological explanation has yet to be identified and confirmed. 

## Conclusions

Clostridium difficile-associated diarrhea (CDAD) is a subset of ADD; however, it accounts only for a small percentage of diarrhea caused by antibiotics. AAD can be caused by multiple other organisms, including Clostridium perfringens, Staphylococcus aureus, Salmonella spp., and Candida. Contrary to the current belief, daptomycin can be associated with severe AAD, necessitating an adjustment of the antibiotic regimen. Clinicians should be mindful of daptomycin-associated diarrhea along with its presentation and treatment. Further studies are needed to identify the pathophysiology of daptomycin-associated diarrhea and other forms of AAD. Understanding its mechanism could help prevent, treat, and reduce the high medical costs associated with antibiotic-adverse events.
